# Deformations of acid-mediated invasive tumors in a model with Allee effect

**DOI:** 10.1007/s00285-025-02209-w

**Published:** 2025-05-05

**Authors:** Paul Carter, Arjen Doelman, Peter van Heijster, Daniel Levy, Philip Maini, Erin Okey, Paige Yeung

**Affiliations:** 1 Department of Mathematics, University of California, Irvine, CA USA; 2 Mathematical Institute, Leiden University, Leiden, Netherlands; 3 Mathematical and Statistical Methods-Biometris, Wageningen University & Research, Wageningen, Netherlands; 4 Program in Applied and Computational Mathematics, Princeton University, Princeton, USA; 5 Mathematical Institute, University of Oxford, Oxford, UK; 6 Division of Applied Mathematics, Brown University, Providence, RI USA; 7 Massachusetts Institute of Technology, Cambridge, USA

**Keywords:** 35C07, 34D15, 35B35, 35B36, 92C50

## Abstract

We consider a Gatenby–Gawlinski-type model of invasive tumors in the presence of an Allee effect. We describe the construction of bistable one-dimensional traveling fronts using singular perturbation techniques in different parameter regimes corresponding to tumor interfaces with, or without, an acellular gap. By extending the front as a planar interface, we perform a stability analysis to long wavelength perturbations transverse to the direction of front propagation and derive a simple stability criterion for the front in two spatial dimensions. In particular we find that in general the presence of the acellular gap indicates transversal instability of the associated planar front, which can lead to complex interfacial dynamics such as the development of finger-like protrusions and/or different invasion speeds.

## Introduction

Evolving cancerous tumors go through several phases. Tumors initially appear as relatively small congregations of cells that have undergone genetic mutations that typically cause the tumor to grow at an abnormally high rate (Wodarz and Komarova 2014). At onset, this growth is regular and sustained by nutrients, etc., that are transported to the tumor by the governing local diffusion processes. However, at a certain stage, this regular mechanism is no longer able to drive the tumor growth process and the tumor enters the next phase in which it invades the surrounding, healthy, tissue. This stage is characterized by a deformation of the surface of the original clump of (tumorous) cells. The nature of the resulting morphology can determine the invasive capacity, and therefore the overall severity, of the tumor. To invade tissue, tumor cells need to overcome the physical barriers of normal cells and extracellular matrix (ECM), the matrix that provides a physical scaffolding for cells. Understanding how tumor cells achieve this is of obvious importance, especially since at the next stage of the process—known as metastasis—the invading surface of the preceding stage may break away from the primary tumor and invade other parts of body to form, often fatal, secondary tumors.

There are many ways to model the multi-scale process of tumor growth and invasion mathematically, ranging from fully discrete to continuum models, and various hybrid models in between—see for instance Anderson and Quaranta (2008); Lowengrub et al. (2010); Turner and Sherratt (2002). Here, we take the continuum point of view and focus on (generalized, nonlinear) reaction-diffusion type models. Several of these models address the role of matrix metalloproteinases (MMPs), which are enzymes secreted by tumor cells that degrade ECM. For example, Perumpanani et al. (1996) proposed a model consisting of six coupled partial differential equations (PDEs) for cell, ECM and MMP densities, where there are cells of different types (phenotypes) and movement is via the process of haptotaxis (movement up adhesive gradients) as well as diffusion. They analysed, in one spatial dimension, the traveling wave behavior of this system, exploring how the wave speed depends on various cell properties. More recently, Katsaounis et al. (2024) developed a hybrid multiscale 3D model employing PDEs and stochastic differential equations in which cell diffusion is modelled nonlinearly. They carry out a numerical study of the system in the context of a number of biologically motivated case studies. In 1996, Gatenby and Gawlinski (1996) published their seminal paper in which they stated their acid-mediated invasion hypothesis. Namely, they proposed that tumor cells, undergoing glycolysis, which produces lactic acid, could invade normal cells due to the acid being more toxic to normal cells than to tumor cells. Their PDE model was composed of three coupled equations, with a cross-dependent degenerate diffusion term for tumor cell movement. This led to the formation of sharp interfaces representing the surface of the growing tumor and the prediction that, in certain cases, an acellular gap would appear between the tumor and normal cell populations. This prediction was validated in the context of head and neck cancer. This model has been extended in a number of studies to account also for ECM degradation (see, for example, Martin et al. 2010, Strobl et al. 2020).

**Fig. 1:**
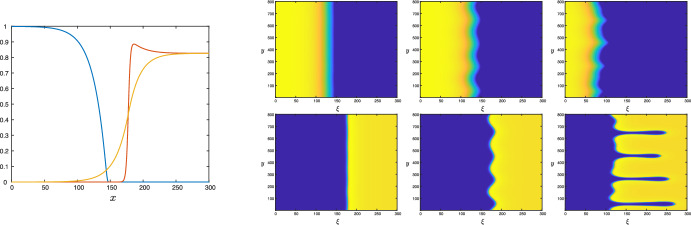
Results of a direct numerical simulation of (1.1) exhibiting the transversal long wavelength instability for parameter values taken from Gatenby and Gawlinski (1996) $(a,\kappa , \delta _1, \delta _2, \delta _3, \rho , \varepsilon ) = (0.35,0.1,12.5,0.1,70.0,1.0, 0.0063)$. In the left panel, the underlying (longitudinally stable) one-dimensional traveling wave is shown (in which the normal cell density *U* is plotted in blue, the tumor cell density *V* in red, and the acid concentration *W* in yellow. The six right panels depict the results of two-dimensional simulations. The initial conditions of the two-dimensional run were constructed by trivially extending this 1D-stable profile in the *y*-direction and adding a small amount of noise. The simulations were performed in a co-moving frame $\xi =x+ct$; in the laboratory frame the tumor would travel with speed $c=0.0401$ to the left, corresponding to the wave speed of the initial front profile (so that in the absence of any instability, the fronts would appear stationary). The corresponding tumor *U*-profiles (top three panels) and *V*-profiles (bottom three panels) are plotted (for $t=20000,30000,40000$ from left to right); for all profiles yellow indicates high density of cells, and blue depicts low density of cells

The mathematical challenges raised by the nonlinear cross-dependent diffusion term in Gatenby and Gawlinski (1996) have inspired many mathematical studies of the traveling wave behavior of this system in one spatial dimension (see Davis et al. 2022; Fasano et al. 2009; Gallay and Mascia 2022 and the references therein). However, these in essence one-dimensional interfaces can only be expected to be stable—and thus observable—in the initial ‘regular’ stage of tumor growth. In the follow-up phase in which the tumor starts to invade the surrounding tissue, the interface most likely will evolve into a structure with a two- or three-dimensional nature. Viewing such shapes as possibly arising from a spatial patterning instability, but departing from the idea of a (growing) tumor surface governed by traveling waves, Chaplain et al. (2001) proposed a Turing-type model composed of growth activating and growth inhibiting chemicals and showed how these gave rise to spatial patterns on a spherical surface which, they proposed, would induce ‘columnar outgrowths of invading cancer cells’. In this paper, we propose a different mechanism for the formation of outgrowths of invading tumor cells that is based on the tumor surface as the interface between tumorous and healthy tissue governed by bistable traveling fronts (see Fig. 1). Namely, we analyse front evolution—or interface dynamics—in a slightly modified form of the Gatenby-Gawlinski (1996) model to explore the potential appearance of transversal instabilities on interfaces that are longitudinally stable. In other words, we consider two-dimensional fronts for which the underlying one-dimensional traveling waves are stable in the direction of propagation, but for which instabilities may form in directions transversal to this direction. In Fig. 1 we show the outcome of a simulation of the (slightly modified) Gatenby-Gawlinski model in two space dimensions that indeed exhibits such a transversal instability: the interface develops ‘fingers’ similar to the phenomenon of viscous fingering in fluid dynamics. We emphasize that the instability develops purely due to extending the (longitudinally stable) front as an interface in two spatial dimensions, in the absence of any deterministic or stochastic forcing or inhomogeneity.

In our modified version of the Gatenby-Gawlinski model (Gatenby and Gawlinski 1996), we assume that tumor cells are not fully resistant to lactic acid by including a death term in the tumor cell equation. We also regularise the nonlinear diffusion term by assuming that normal cells do not form an impenetrable barrier to tumor cell invasion. While we do this primarily for mathematical convenience, it should be noted that there is a biological justification for this as some tumor cells upregulate the expression of aquaporins (see the review Verkman et al. 2008) which, it has been hypothesised, may allow cells to ‘bulldoze’ their way through surrounding cells and tissues (McLennan et al. 2020). Lastly, we replace the logistic growth term for cancer cells by a term that allows for the Allee effect, which has been proposed to play a role in cancer tumor growth dynamics similar to in ecosystems—see Gerlee et al. (2022); Johnson et al. (2019); Korolev et al. (2014); Sewalt et al. (2016) and the references therein. Thus, we consider the following reaction-diffusion model for tumor invasion,

1.1 $$\begin{aligned} \begin{aligned} U_\tau&=U(1-U)-\delta _1 UW\,,\\ V_\tau&= \rho V(1-V)(V-a)-\delta _2 VW+\nabla \cdot ((1+\kappa -U) \nabla V)\,,\\ W_\tau&= \delta _3(V-W)+\frac{1}{\varepsilon ^2}\Delta W\,, \end{aligned} \end{aligned}$$

where $(U,V,W)(x,y,\tau )$ represent normal cell density, tumor cell density, and acid (concentration) produced by the tumor cells, respectively, at spatial position $(x,y)\in \mathbb {R}^2$ and time $\tau \in \mathbb {R}^+$. The parameter $\delta _1$ measures the destructive influence of acid on healthy tissue and can thus be seen as an indicator of tumor aggressivity, the new parameter $\delta _2$ represents the impact of acid on the tumor itself and we assume that $0\le \delta _2 <\delta _1$, i.e. the effect of *W* on *V* is less than that of *W* on *U* (but not necessarily zero). The parameter $\rho > 0$ measures the relative production rate of tumor cells compared to healthy cells and $0<a<1$ the relative strength of the Allee effect. Since the diffusive spreading speed of acid is much faster than that of cells (Gatenby and Gawlinski 1996) it follows that $0< \varepsilon \ll 1$ so that reaction-diffusion system (1.1) is singularly perturbed. Finally, the parameter $\kappa$ measures the regularized nonlinear diffusion effect and is assumed to satisfy $0<\varepsilon \ll \kappa \ll 1$.

In the spirit of Korolev et al. (2014), the present paper was motivated by recent progress on the formation of invasive, ‘fingering’, interface patterns as in Fig. 1 in the context of coexistence patterns (between grasslands and bare soil) in dryland ecosystems (Carter 2024; Carter et al. 2023; Fernandez-Oto et al. 2019). In fact, in Carter (2024) and Carter et al. (2023) criteria have been developed by which the transversal (in)stability of planar interfaces in a general class of singularly perturbed two-component reaction-diffusion equations—that includes typical dryland ecosystem models—can be determined. The present model does not directly fall into this class of systems: (1.1) contains three components and has a nonlinear diffusion term—unlike the systems considered in Carter et al. (2023). Nevertheless, we show in this paper that the methods by which the criteria in Carter et al. (2023) are deduced can also be applied to the present modified Gatenby-Gawlinski model.

**Fig. 2:**
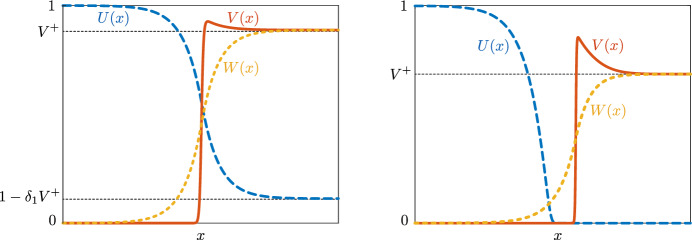
Bistable traveling front solutions in the benign (left) and malignant (right) cases. Note that in the malignant case, an acellular gap may appear in between the interfaces formed by the *U* and *V* profiles and that this case corresponds to the situation of Fig. 1

As in Carter et al. (2023), the transversal (in)stability results are derived from a detailed investigation into the structure of the (one-dimensional) traveling front on which the evolving two-dimensional interface is based (see Fig. 1). These fronts correspond to heteroclinic connections between stable background states—or homogeneous equilibria—in (1.1). The background states of (1.1) are given by

1.2 $$\begin{aligned} P_1 = (0,0,0), \quad P_2 = (1,0,0), \quad P_3^\pm = (1-\delta _1 V^\pm , V^\pm , V^\pm ), \quad P_4^\pm =(0,V^\pm , V^\pm ), \end{aligned}$$

with

1.3 $$\begin{aligned} V^\pm = \frac{\rho (1+a)-\delta _2 \pm \sqrt{\left( \rho (1+a)-\delta _2\right) ^2-4\rho ^2a}}{2\rho }, \end{aligned}$$

where we note that the states $P_3^\pm$ only are biologically relevant for parameter combinations such that $V^\pm$ are real and positive and $\delta _1V^+<1$, and $P_4^\pm$ for parameters such that $V^\pm > 0$. It follows from linear stability analysis (see Appendix A) that $P_2$ is stable, while $P_1, P_3^-, P_4^-$ are unstable. The background state $P_3^+$ is stable for $\delta _1V^+<1$, while $P_4^+$ is stable if $\delta _1V^+>1$. In this paper, we refer to the former as the *benign* case, and the latter as the *malignant* case, based on whether normal cells *U* can coexist with tumor cells *V* in the nontrivial stable steady state. In the benign case $\delta _1V^+<1$ we will construct (and further study) a bistable traveling front between homogeneous background states $P_2$ and $P_3^+$, while in the malignant case $\delta _1V^+>1$, we will consider a bistable front between $P_2$ and $P_4^+$—see Fig. 2.

We construct three types of bistable traveling fronts/tumor interfaces using geometric singular perturbation theory: a benign front, a malignant no-gap front and a malignant gap front (see upcoming Fig. 6 for the distinct geometries of these cases). The overall geometry of the construction of fronts is similar to that of Davis et al. (2022), in which invasion fronts were constructed in the model (1.1) in the absence of the Allee effect. Some differences arise here due to the presence of the Allee effect, which results in bistable fronts existing at a unique wave speed, as opposed to a range of speeds. These bistable fronts are more amenable to a two-dimensional stability analysis, as the critical spectrum associated with such fronts in one spatial dimension takes the form of a single eigenvalue at $\lambda =0$ due to translation invariance. As in Carter et al. (2023), we do not explicitly analyze the longitudinal, i.e. one-dimensional (in the direction of propagation), stability of these traveling fronts: we assume that they are longitudinally stable and thus that the translational eigenvalue at $\lambda = 0$ is the most critical eigenvalue. Note that the assumptions are based on numerical observations and especially on numerical evaluations of the spectrum associated with the linearized stability problem (cf. Sect.4). The stability of the associated planar interface spanned by the one-dimensional fronts can be determined by an additional transversal Fourier expansion—parameterized by wavenumber $\ell \in \mathbb {R}$. As a consequence, the translational eigenvalue re-appears as a local extremum at $\ell = 0$ of a critical curve $\lambda _\textrm{c}(\ell )$, i.e. $\lambda _\textrm{c}(0) = 0$. Observe that this curve is symmetric in $\ell$. Extending the methods developed in Carter et al. (2023), we derive an explicit approximation of $\lambda _\textrm{c}''(0)$, where prime denotes differentiation with respect to $\ell$. Clearly, the interface is unstable with respect to transversal long wavelength perturbations if $\lambda _\textrm{c}''(0) > 0$ (so that $\lambda _\textrm{c}(\ell ) > 0$ for $\ell$ sufficiently small). In that case, the originally planar interface will be unstable and growing long wavelength spatial structures will emerge from the interface. Numerical simulations show that the interface may develop protruding fingers (Fig. 1) or cusps (see Sect. 4), which is similar to the observations of evolving interfaces (between grasslands and bare soil) in dryland ecosystems under the same circumstances (i.e. also with $\lambda _\textrm{c}''(0) > 0$) (Carter 2024; Carter et al. 2023; Fernandez-Oto et al. 2019).

The explicit character of our analysis establishes a direct correspondence between the nature—benign, malignant no-gap/gap—of the underlying traveling front and the initiation of transversal instabilities of the evolving interface between tumorous and regular cells. We briefly summarize some of our main findings (and refer to Sect. 4 for the more extensive discussion): Firstly, we deduce directly from our derivation of $\lambda _\textrm{c}''(0)$, and from the geometry of the associated front, that the presence of the acellular gap immediately implies transversal instability of the tumor interface in this model for sufficiently small $\varepsilon >0$. In the benign and malignant no-gap cases, however, the tumor interface can be stable or unstable to long wavelength perturbations depending on parameters. Our characterization of this instability in terms of the coefficient $\lambda _\textrm{c}''(0)$ allows us to easily determine stability boundaries in parameter space via numerical continuation; see Sect. 4. We also explore the effect of individual parameters, such as the parameter $\delta _1$, which turns out to be a natural parameter to transition between the three cases of benign and malignant no-gap/gap fronts, and the Allee parameter *a*. In particular, for the latter, we find that in general a stronger Allee effect, i.e. larger *a*, leads to a decrease in the speed of the associated front, but also leads to the onset of the transversal instability.

The remainder of the paper is organized as follows. In Sect. 2, we analyze the traveling wave equation associated with (1.1) to describe the existence construction of traveling fronts in the singular limit $\varepsilon \rightarrow 0$. Using formal singular perturbation arguments we examine the transversal long wavelength (in)stability of these fronts in Sect. 3, and we conclude with numerical simulations and a discussion in Sect. 4.

## Existence of traveling fronts

In this section, we show that (1.1) supports traveling fronts. We will largely follow the geometric singular perturbation theory approach of Davis et al. (2022). In Davis et al. (2022) the formal asymptotic results of Holder et al. (2014) on the original nondimensionalized Gatenby-Gawlinski model were proven rigorously and a geometric interpretation of the benign and malignant cases, as well as the acellular gap, were given. As the focus of the current manuscript is largely on the stability of the traveling fronts, see Sect. 3, and since the derivation of the existence results for the current setting largely mimics the approaches and proofs of Davis et al. (2022), we only succinctly derive the results (and we refer to Davis et al. (2022) for the rigorous constructions in a similar setting).

We set $(U,V,W)(x,y,\tau ) = (u,v,w)(x+\varepsilon ^\nu c\tau )$ and search for traveling waves in the traveling coordinate $\xi := x+\varepsilon ^\nu c\tau$, where $c>0$. This results in a singularly perturbed traveling wave ordinary differential equation (ODE)

2.1 $$\begin{aligned} \begin{aligned} \varepsilon ^\nu cu'&=u(1-u)-\delta _1 uw\,,\\ \varepsilon ^\nu cv'&= \rho v(1-v)(v-a)-\delta _2 vw+((1+\kappa -u) v')'\,,\\ \varepsilon ^\nu cw'&= \delta _3(v-w)+\frac{1}{\varepsilon ^2}w''\,, \end{aligned} \end{aligned}$$

where $'$ means differentiation with respect to $\xi$. Upon introducing^1^$q:= \varepsilon ^{-\nu }(1+\kappa -u) v' - cv$ and $p= w'/\varepsilon$, we rewrite this system as a first order slow/fast ODE

2.2 $$\begin{aligned} \begin{aligned} \varepsilon ^\nu u'&=\frac{u}{c}\left( 1-u-\delta _1w\right) \,,\\ v'&= \varepsilon ^\nu \frac{q+c v}{1+\kappa -u}\,,\\ \varepsilon ^\nu q'&= -\rho v(1-v)(v-a)+\delta _2 vw\,,\\ w'&= \varepsilon p\,,\\ p'&= \varepsilon \left( c\varepsilon ^{\nu +1} p-\delta _3(v-w)\right) \,. \end{aligned} \end{aligned}$$

Observe that the *v*-equation of (2.2) is not singular due to the regularisation term $\kappa$ and the fact that we are looking for traveling fronts with normal cell density *u* between 0 and 1. The fixed points

$$\begin{aligned}&p_1 = (0,0,0,0,0), \quad p_2 = (1,0,0,0,0), \\&p_3^\pm = (1-\delta _1 V^\pm , V^\pm ,c V^\pm , V^\pm ,0), \quad p_4^\pm =(0,V^\pm , c V^\pm ,V^\pm ,0), \end{aligned}$$

of (2.2) in (*u*, *v*, *q*, *w*, *p*)-space correspond to the steady states $P_1,P_2,P_3^\pm , P_4^\pm$ (1.2) of (1.1).

We observe three critical $\nu$-cases in (2.2): $\nu =0$ and $\nu =\pm 1$. In Holder et al. (2014) it was shown for the original nondimensionalized Gatenby-Gawlinski model that, when translated to the current setting, the case $\nu =1$ does not lead to traveling fronts. In contrast, the case $\nu =-1$ led to *fast traveling fronts*, while the case $\nu =0$ led to *slow traveling fronts*. In Davis et al. (2022) both the fast traveling fronts and slow traveling fronts were investigated. Based on our numerical simulations it appears that the $\nu =0$-case is the most relevant for the instabilities we want to study. Therefore, we focus on the $\nu =0$-case and only briefly highlight the $\nu =-1$-case in the remark below.

For $\nu =0$ in (2.2) we observe that (*u*, *v*, *q*) are *fast variables*, while (*w*, *p*) are *slow variables*. Letting $\varepsilon \rightarrow 0$, we find the critical manifolds

**Fig. 3:**
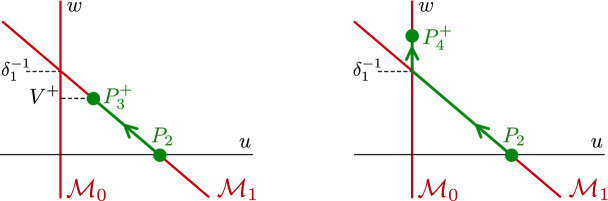
Schematic of the singular heteroclinic orbit in the benign case where $u \ne 0$ after the invasion of the traveling wave (left) and the malignant case where $u = 0$ after the invasion (right)

2.3 $$\begin{aligned} \begin{aligned} \mathcal {M}_0&= \left\{ (u,v,q,w,p) \quad | \quad u=0,\,\, q=-cv,\,\, \rho v(1-v)(v-a) = \delta _2vw \right\} \,,\\ \mathcal {M}_1&= \left\{ (u,v,q,w,p) \quad | \quad u=1-\delta _1 w,\,\, q=-cv,\,\, \rho v(1-v)(v-a) = \delta _2 vw \right\} \,, \end{aligned} \end{aligned}$$

which meet along the nonhyperbolic transcritical singularity curve at $w = 1/\delta _1$. The fixed points $p_1,p_4^\pm$ lie on $\mathcal {M}_0$, while $p_2, p_3^\pm$ lie on $\mathcal {M}_1$. In the benign case $\delta _1 V^+<1$, see (1.3), we can construct a singular heteroclinic orbit between $p_2$ and $p_3^+$ in the subspace $u=1-\delta _1 w$, that is, the orbit is entirely contained within $\mathcal {M}_1$ and avoids the transcritical singularity; see Fig. 3. In the malignant case $\delta _1 V^+>1$, we can construct a singular heteroclinic orbit between $p_2$ and $p_4^+$, but it necessarily passes through the transcritical curve in order to transition from $\mathcal {M}_1$ to $\mathcal {M}_0$.

In order to construct singular heteroclinic orbits in each case, we consider the associated layer problems describing the dynamics near the interface (the *fast field*) and reduced problems describing the dynamics away from the interface (the *slow fields*) in the next sections.

### Remark 2.1

For the fast traveling fronts with $\nu =-1$, the singularly perturbed problem reduces, upon rescaling space once more, at leading order to

$$\begin{aligned} \begin{aligned} \dot{u}&=\frac{u}{c}\left( 1-u-\delta _1w\right) \,,\\ \dot{q}&= - \rho v(1-v)(v-a)+\varepsilon ^2 \delta _2 vw\,,\\ \dot{w}&= p\,,\\ \dot{p}&= c p-\delta _3(v-w)\,, \end{aligned} \end{aligned}$$

where ^.^ denotes differentiation with respect to $\bar{\xi }:=\varepsilon x+ct$, on the attracting four dimensional critical manifold

$$ \mathcal {M}_0^F:= \left\{ (u,v,q,w,p) \,\, \left| \,\, v = -\dfrac{q}{c}\right. \right\} \,. $$

That is, the existence of fast traveling fronts boils down to showing the existence of heteroclinic solutions to

$$\begin{aligned} \begin{aligned} \dot{u}&=\frac{u}{c}\left( 1-u-\delta _1w\right) \,,\\ c \dot{v}&= \rho v(1-v)(v-a)-\delta _2 vw\,,\\ \ddot{w} - c \dot{w}&= -\delta _3(v-w)\,. \end{aligned} \end{aligned}$$

In this manuscript, we do not pursue this direction any further; however, see Theorem 1.1 of Davis et al. (2022).

### Layer problem

We consider the layer problem describing the dynamics near the interface (the fast field) for $\nu =0$. That is, we set $\nu =0$ in (2.2) and take the singular limit $\varepsilon \rightarrow 0$ to obtain

2.4 $$\begin{aligned} \begin{aligned} u'&=\dfrac{u}{c}\left( 1-u-\delta _1w\right) \,,\\ v'&= \dfrac{q+cv}{1+\kappa -u}\,,\\ q'&= -\rho v(1-v)(v-a)+\delta _2 vw\,,\\ \end{aligned} \end{aligned}$$

where we recall that $'$ denotes differentiation with respect to $\xi =x+ct$, with the fast variables *w* and *p* fixed constants. The fixed points of (2.4) are related to the critical manifolds $\mathcal {M}_{0,1}$ (2.3), and note that these have several branches

$$\begin{aligned} \mathcal {M}^0_0&= \left\{ (u,v,q,w,p) \,\, | \,\, u=v=q=0\right\} \,,\\ \mathcal {M}^\pm _0&= \left\{ (u,v,q,w,p) \,\, | \,\, u=0,\,\, v = v^\pm (w),\,\, q=-c v^\pm (w) \right\} \,,\\ \mathcal {M}^0_1&= \left\{ (u,v,q,w,p) \,\, | \,\, u=1-\delta _1 w,\,\, v=q=0\right\} \,,\\ \mathcal {M}^\pm _1&= \left\{ (u,v,q,w,p) \,\, | \,\, u=1-\delta _1 w,\,\, v = v^\pm (w),\,\, q=- c v^\pm (w) \right\} \,, \end{aligned}$$

where

$$\begin{aligned} v^\pm (w) = \frac{1+a\pm \sqrt{(1-a)^2-4\delta _2 w/\rho }}{2}\,. \end{aligned}$$

We note that the fixed point $p_2$ lies on $\mathcal {M}^0_1$, while $p_3^+$ lies on $\mathcal {M}^+_1$, and $p_4^+$ lies on $\mathcal {M}_0^+$. By inspecting the Jacobian matrix of (2.4)

$$\begin{aligned} J = \begin{pmatrix} \frac{1}{c}\left( 1-2u-\delta _1 w\right) & 0 & 0\\ \frac{q+cv}{(1+\kappa -u)^2} & \frac{c}{1+\kappa -u} & \frac{1}{1+\kappa -u} \\ 0 & \rho (3v^2-2av+a) +\delta _2 w& 0 \end{pmatrix} \end{aligned}$$

and noting the lower block triangular structure of this matrix, we see that the critical manifolds all lose normal hyperbolicity along the transcritical curve $w=\delta _1^{-1}$ where $1-2u- \delta _1 w=0$ and along the fold curve where $v^+(w)=v^-(w)$, or equivalently $\rho (3v^2-2av+a) +\delta _2 w=0$. Away from these curves, the manifolds $\mathcal {M}^0_0$, $\mathcal {M}^0_1$, $\mathcal {M}^+_0$, and $\mathcal {M}^+_1$ are all normally hyperbolic and of saddle type in the (*v*, *q*)-subsystem. The manifolds $\mathcal {M}^-_0$ and $\mathcal {M}^-_1$ are of center type when $c=0$ and normally repelling/attracting in the (*v*, *q*)-subsystem when $c\lessgtr 0$; however these two manifolds will not be important for the analysis.

**Fig. 4:**
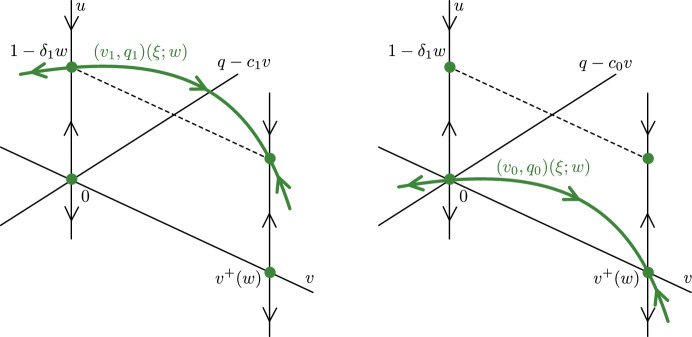
(Left) The fast orbit $(v_1,q_1)(\xi ;w)$ in the subspace $u=1-\delta _1 w$ with $0 \le w < \min \{\rho (1-a)^2/(4\delta _2),1/\delta _1\}$. (Right) The fast orbit $(v_0,q_0)(\xi ;w)$ in the subspace $u=0$ with $w > 0$

Importantly, for the benign case $u=1-\delta _1 w$ in the layer dynamics and for each $0\le w < \min \{\rho (1-a)^2/(4\delta _2),1/\delta _1\}$ (within this subspace $u=1-\delta _1 w$) there exists a heteroclinic orbit $(v,q)=(v_1,q_1)(\xi ;w)$ between $\mathcal {M}^0_1$ and $\mathcal {M}^+_1$ satisfying the planar ODE

$$\begin{aligned} \begin{aligned} v'&= \frac{q+cv}{\kappa +\delta _1 w}\,,\\ q'&= -\rho v(1-v)(v-a)+\delta _2 vw\,,\\ \end{aligned} \end{aligned}$$

with $c = c_1(w)$, see Fig. 4. Here, $v_1(\xi ;w)$ and $c_1(w)$ are given by

$$\begin{aligned} v_1(\xi ;w)&:= \frac{v^+(w)}{2}\left( 1+\tanh \left( \frac{v^+(w)}{2}\sqrt{\frac{\rho }{2(\kappa +\delta _1w)}} \xi \right) \right) \,, \\ c_1(w)&:=\sqrt{\frac{2(\kappa +\delta _1w)}{\rho }}\left( \frac{v^+(w)}{2}-v^-(w)\right) . \end{aligned}$$

Similarly, in the subspace $u=0$, there exists a heteroclinic orbit $(v,q)=(v_0,q_0)(\xi ;w)$ between $\mathcal {M}^0_0$ and $\mathcal {M}^+_0$ satisfying the planar ODE

$$\begin{aligned} \begin{aligned} v'&= \frac{q+cv}{1+\kappa }\,,\\ q'&= -\rho v(1-v)(v-a)+\delta _2 vw\,,\\ \end{aligned} \end{aligned}$$

for $$c = c_0(w)$$, where

$$\begin{aligned} v_0(\xi ;w)&:= \frac{v^+(w)}{2}\left( 1+\tanh \left( \frac{v^+(w)}{2}\sqrt{\frac{\rho }{2(1+\kappa )}} \xi \right) \right) \,, \\ c_0(w)&:=\sqrt{\frac{2(1+\kappa )}{\rho }}\left( \frac{v^+(w)}{2}-v^-(w)\right) . \end{aligned}$$

### Reduced problem

Rescaling $\zeta =\varepsilon \xi$ and setting $\varepsilon =0$, we obtain the reduced problem describing the dynamics away from the interface (the slow fields)

$$\begin{aligned} \begin{aligned} w_\zeta&= p\,,\\ p_\zeta&= -\delta _3(v-w)\,, \end{aligned} \end{aligned}$$

which does not explicitly depend on *u* and describes the leading order dynamics on the critical manifolds. When restricted to $\mathcal {M}^0_0$ or $\mathcal {M}_1^0$, where $v=0$, this results in the system

2.5 $$\begin{aligned} \begin{aligned} w_\zeta&= p\,,\\ p_\zeta&= \delta _3 w\,, \end{aligned} \end{aligned}$$

while on $\mathcal {M}^+_0$ and $\mathcal {M}^+_1$, where $v=v^+(w)$, we have the system

2.6 $$\begin{aligned} \begin{aligned} w_\zeta&= p\,,\\ p_\zeta&= \delta _3(w-v^+(w))\,. \end{aligned} \end{aligned}$$

The system (2.5) admits a saddle equilibrium at (0, 0) corresponding to the fixed point $p_2$ of the full system, while (2.6) admits a saddle equilibrium at $(V^+,0)$, see (1.3), corresponding to $p_3^+$ or $p_4^+$. The (un)stable manifolds $\mathcal {W}^\textrm{s,u}(0,0)$ of the equilibrium (0, 0) of (2.5) within $\mathcal {M}^0_0$ or $\mathcal {M}^0_1$ are given by the lines $\{p = \pm \sqrt{\delta _3}w\}$, while the (un)stable manifolds $\mathcal {W}^\textrm{s,u}(V^+,0)$ of the equilibrium $(V^+,0)$ of (2.6) within $\mathcal {M}^+_0$ or $\mathcal {M}^+_1$ can be determined (implicitly) from the Hamiltonian structure of (2.6). In particular, the quantities

$$\begin{aligned} E_0(w,p):=\frac{1}{2}p^2-\frac{\delta _3}{2}w^2\,, \end{aligned}$$

and

$$\begin{aligned} E_+(w,p):= E_0(w,p) + E(w) = \frac{1}{2}p^2-\frac{\delta _3}{2}w^2+\frac{\delta _3}{2}(V^+)^2+\int _{V^+}^w\delta _3v^+(s)\textrm{d}s\,, \end{aligned}$$

are conserved in (2.5) and (2.6), respectively, and satisfy

$$\begin{aligned} E_0(0,0)=0=E_+(V^+,0)\,. \end{aligned}$$

**Fig. 5:**
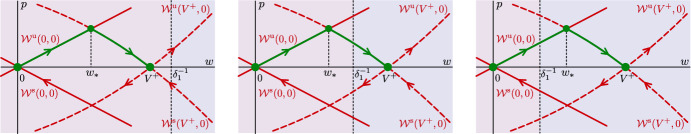
The slow orbits on $\mathcal {M}^0_{0,1}$ and $\mathcal {M}^+_{0,1}$, corresponding to $\mathcal {W}^\textrm{u}(0,0)$ and $\mathcal {W}^\textrm{u}(V^+,0)$, respectively, in the benign (left), malignant no-gap (center), and malignant gap (right) cases. The manifolds $\mathcal {W}^\textrm{s,u}(0,0)$ on $\mathcal {M}^0_{0,1}$ are depicted in solid red, while the manifolds $\mathcal {W}^\textrm{s,u}(V^+,0)$ on $\mathcal {M}^+_{0,1}$ are depicted in dashed red, and the vertical dashed line indicates the subspace $w=\delta _1^{-1}$ at which the reduced dynamics transition from $\mathcal {M}^{0,+}_{0}$ to $\mathcal {M}^{0,+}_{1}$; see also Fig. 6

The equations (2.5) and (2.6) only differ by the term $\delta _3 v^+(w)$ in *p*-component and this term is always strictly positive. Furthermore, the flow of (2.6) through the line segment $\{(w,p)=(0,0)+t(V^+,0)\,|\,t \in (0,1)\}$ is downwards, while the flow through $\{(w,p)=(V^+,0)+t( 0,\sqrt{\delta _3} V^+)\,|\,t \in (0,1)\}$ points to the right. Hence the projection of the unstable manifold $\mathcal {W}^\textrm{u}(0,0)$ from $\mathcal {M}^0_1$ onto $\mathcal {M}^+_1$ transversely intersects the stable manifold $\mathcal {W}^\textrm{s}(V^+,0)$ of the equilibrium $(V^+,0)$ at some $(w,p) = (w_*, p_*)$, where $p_* = \sqrt{\delta _3}w_*$, and $0<w_*<V^+$ satisfies

$$\begin{aligned} E_+(w_*,\sqrt{\delta _3}w_*)=0\,, \end{aligned}$$

or equivalently,

2.7 $$\begin{aligned} 0=(V^+)^2+\int _{V^+}^{w_*}\left( 1+a+\sqrt{(1-a)^2-\frac{4\delta _2 z}{\rho }}\right) \textrm{d}z \,, \end{aligned}$$

see Fig. 5. The same holds regarding the projection of the unstable manifold $\mathcal {W}^\textrm{u}(0,0)$ from $\mathcal {M}^0_0$ onto $\mathcal {M}^+_0$, which transversely intersects the stable manifold $\mathcal {W}^\textrm{s}(W^+,0)$ of the equilibrium $(W^+,0)$ at $w=w_*$.

### Singular heteroclinic orbits

Combining orbits from the reduced and layer problems, we can construct singular heteroclinic orbits in the benign ($\delta _1 V^+<1$) and malignant ($\delta _1 V^+>1$) cases.

**Fig. 6:**
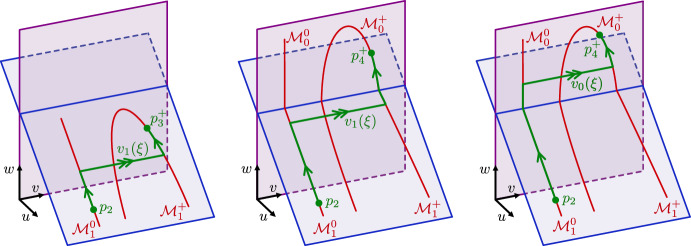
Structure of the singular heteroclinic orbit in the benign (left), malignant no-gap (middle), and malignant gap (right) cases. The subspace $\{u=0\}$ is depicted in purple, while the subspace $\{u=1-\delta _1 w\}$ is depicted in blue

Benign case: In the benign case, we construct a singular heteroclinic orbit between $p_2$ and $p^+_3$ by concatenating the three orbit segments, see Fig. 5 (left panel) and Fig. 6 (left panel):

1. slow orbit on $\mathcal {M}^0_1$ given by $\mathcal {W}^\textrm{u}(0,0)\cap \{0\le w\le w_*\}$, with $w^*<V^+$.
2. fast jump $(v_*,q_*)(\xi ):=(v_1,q_1)(\xi ;w_*)$ with speed $c=c_*(w):=c_1(w_*)$ in the layer problem (2.4) for $w=w_*$ in the subspace $u = 1-\delta _1w_*$, where $w_*$ is defined as in (2.7), see Fig. 4. Note that $\delta _1w_*<1$ in the benign case.
3. slow orbit on $\mathcal {M}^+_1$ given by $\mathcal {W}^\textrm{s}(V^+,0)\cap \{w_*< w\le V^+\}$.

In the benign case, $\mathcal {M}^0_1$ and $\mathcal {M}^+_1$ are normally hyperbolic in the relevant region, and the fast jump is transversely constructed (with the speed $c\approx c_1(w_*)$ as a free parameter). Therefore, we expect that the singular orbit perturbs to a traveling front of the full problem for $0<\varepsilon \ll 1$.

Malignant case: In the malignant case, we construct a heteroclinic orbit between $p_2$ and $p^+_4$, which necessarily crosses through the transcritical curve $w = \delta _1^{-1}$, see Fig. 3. However, we must split this into a further two cases, namely whether the corresponding value of $w_*$ (as defined in (2.7)) satisfies $w_*\lessgtr \delta _1^{-1}$, as this determines whether the fast jump in the layer problem (2.4) occurs in the subspace $u=0$ or $u=1-\delta _1w$. That is, whether the fast jump occurs *before* of *after* crossing the transcritical curve. In the latter case $w_*>\delta _1^{-1}$, there is a region in which the values of both *u*, *v* are zero, *before* the fast jump occurs, that is, the front admits a gap, called the acellular gap, where only acid is present. In the former case $w_*<\delta _1^{-1}$ there is no acellular gap (as in the benign case above). The boundary between these cases in parameter space is determined by the condition

$$\begin{aligned} 0=(V^+)^2+\int _{V^+}^{\delta _1^{-1}}1+a+\sqrt{(1-a)^2-\frac{4\delta _2 z}{\rho }}\textrm{d}z \,, \end{aligned}$$

where $V^+$ is as in (1.3). We refer to these (sub)cases as the malignant gap and malignant no-gap cases, respectively.

In the **malignant no-gap case** $w_*<\delta _1^{-1}$, we have the following singular concatenation, see Fig. 5 (middle panel) and Fig. 6 (middle panel):

1. slow orbit on $\mathcal {M}^0_1$ given by $\mathcal {W}^\textrm{u}(0,0)\cap \{0\le w\le w_*\}$.
2. fast jump $(v_*,q_*)(\xi ):=(v_1,q_1)(\xi ;w_*)$ with speed $c=c_*(w):=c_1(w_*)$ in the layer problem (2.4) for $w=w_*$ in the subspace $u = 1-\delta _1w_*$, where $w_*$ is defined as in (2.7), see Fig. 4. Note that $\delta _1w_*<1$ in the malignant no-gap case.
3. slow orbit on $\mathcal {M}^+_1$ given by $\mathcal {W}^\textrm{s}(V^+,0)\cap \{w_*< w\le \delta _1^{-1}\}$.
4. slow orbit on $\mathcal {M}^+_0$ given by $\mathcal {W}^\textrm{s}(V^+,0)\cap \{\delta _1^{-1}\le w< V^+\}$.

In the **malignant gap case** $w_*>\delta _1^{-1}$, we have the concatenation, see Fig. 5 (right panel) and Fig. 6 (right panel):

1. slow orbit on $\mathcal {M}^0_1$ given by $\mathcal {W}^\textrm{u}(0,0)\cap \{0\le w\le \delta _1^{-1}\}$.
2. slow orbit on $\mathcal {M}^0_0$ given by $\mathcal {W}^\textrm{u}(0,0)\cap \{\delta _1^{-1}< w\le w_*\}$.
3. fast jump $(v_*,q_*)(\xi ):=(v_0,q_0)(\xi ;w_*)$ with speed $c=c_*(w):=c_0(w_*)$ in the layer problem (2.4) for $w=w_*$ in the subspace $u = 0$, where $w_*$ is defined as in (2.7), see Fig. 4. Note that $\delta _1w_*>1$ in this case.
4. slow orbit on $\mathcal {M}^+_0$ given by $\mathcal {W}^\textrm{s}(V^+,0)\cap \{w_*< w\le W^+\}$.

**Fig. 7:**
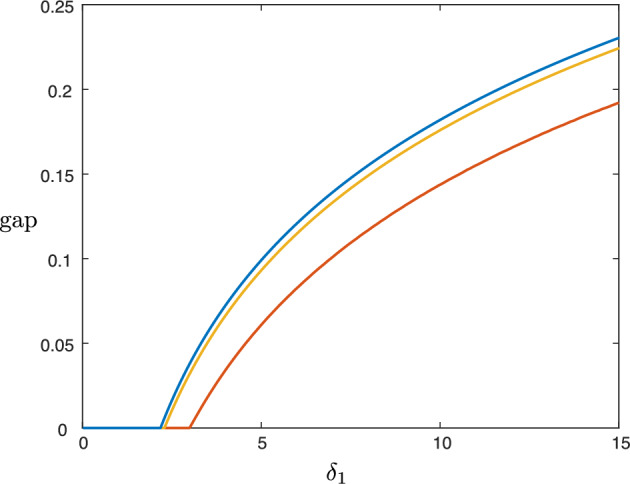
Plot of gap width versus $\delta _1 \in (0.1,15)$ obtained by numerical continuation of the traveling wave equation (2.2) for the parameter values $(a,\kappa , \delta _2,\delta _3, \rho ) = (0.1,0.1,0.1,70,1.0)$ and $\varepsilon =0.0063$ (red), $\varepsilon =10^{-4}$ (yellow). The gap width was computed by measuring the spatial width where both the *u* and *v* profiles of the corresponding front solution were below a threshold value of $10\varepsilon$. Also plotted (blue) is the singular limit gap width obtained by solving (2.7) for $w_*$ using Mathematica and integrating (2.5) to obtain the time spent along $\mathcal {M}^0_0$ between $w=\delta _1^{-1}$ and $w=w_*$

The acellular gap manifests as the slow orbit portion $\mathcal {W}^\textrm{u}(0,0)\cap \{\delta _1^{-1}\le w\le w_*\}$ on $\mathcal {M}^0_0$. In general, one expects the gap size to thus increase with $\delta _1$; see Fig. 7.

In both cases (malignant gap and malignant no-gap), the singular orbit traverses the transcritical curve $\delta _1w=1$ along a slow orbit. In the critical crossover case $w_*=\delta _1^{-1}$, the fast jump occurs precisely along the transcritical singularity curve. Due to the lack of hyperbolicity which occurs at the transcritical bifurcation, the persistence of orbits in the malignant case for $0<\varepsilon \ll 1$ is nontrivial, necessitating the use of blow-up desingularization methods (Krupa and Szmolyan 2001). Nevertheless we assume their persistence for sufficiently small $\varepsilon >0$.

## Stability of planar tumor interfaces

**Fig. 8:**
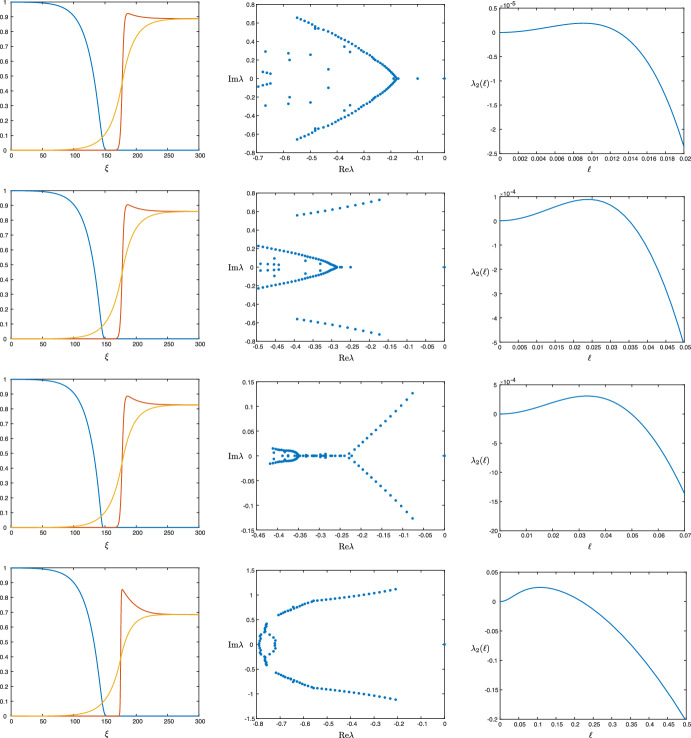
1D traveling wave profiles obtained for the parameter values $(a,\kappa , \delta _1, \delta _2, \delta _3, \rho , \varepsilon ) = (0.1,0.1,12.5,0.1,70,1, 0.0063)$ (first row), $(a,\kappa , \delta _1, \delta _2, \delta _3, \rho , \varepsilon ) = (0.25,0.1,12.5,0.1,70,1, 0.0063)$ (second row), and $(a,\kappa , \delta _1, \delta _2, \delta _3, \rho , \varepsilon ) = (0.35,0.1,12.5,0.1,70,1, 0.0063)$ (third row), $(a,\kappa , \delta _1, \delta _2, \delta _3, \rho , \varepsilon ) = (0.25,0.05,11.5,3,1,15, 0.05)$ (fourth row). The *u*, *v*, *w* profiles are plotted in blue, red, yellow, respectively. Profiles were obtained by solving the traveling wave equation (2.1) in MATLAB. Also shown are the 1D spectra, providing numerical evidence that all four solutions are 1D-stable, as well as a continuation of the critical eigenvalue $\lambda _\textrm{c}(\ell )$ for small, positive values of the wavenumber $\ell$

Given a traveling front $(u_\textrm{h},v_\textrm{h},w_\textrm{h})(\xi )$ with speed $c_\textrm{h} = c_*(w_*)+\mathcal {O}(\varepsilon )$ constructed using the slow/fast structure as in the preceding section, we now consider its spectral stability in two space dimensions, focusing on the long wavelength (in)stability criterion as explored in Carter et al. (2023). Thus, like in Carter et al. (2023), we do not explicitly analyze the stability of the front $(u_\textrm{h},v_\textrm{h},w_\textrm{h})(\xi )$ with respect to longitudinal perturbations, i.e. perturbations that only depend on $\xi$ (or *x*). If the front is longitudinally stable—as is strongly suggested by our numerical results—then the upcoming stability criterion determines the (in)stability of the front to long wavelength perturbations in the *y*-direction, i.e. transverse to the direction of propagation. (We again refer to Carter et al. (2023) for a review of the literature on methods by which the longitudinal (in)stability of a singular traveling front as $(u_\textrm{h},v_\textrm{h},w_\textrm{h})(\xi )$ can be established analytically.) We also refer ahead to Sect. 4 for numerical evidence (see Fig. 8) that the traveling fronts under consideration are 1D spectrally stable.

### Long wavelength (in)stability

In a comoving frame, we rewrite (1.1) as

3.1 $$\begin{aligned} \begin{aligned} U_\tau&=F(U,W)-c U_\xi \,,\\ V_\tau&= G(U,V,W)+\nabla \cdot ((1+\kappa -U)\nabla V)-c V_\xi \,,\\ W_\tau&= H(V,W)+\frac{1}{\varepsilon ^2}\Delta W -c W_\xi \,, \end{aligned} \end{aligned}$$

where

3.2 $$\begin{aligned} F(U,W)&=U(1-U)-\delta _1 UW \,,\end{aligned}$$

3.3 $$\begin{aligned} G(U,V,W)&=\rho V(1-V)(V-a)-\delta _2 VW\,,\end{aligned}$$

3.4 $$\begin{aligned} H(V,W)&=\delta _3(V-W)\,, \end{aligned}$$

and $(U,V,W)=(U,V,W)(\xi ,y,\tau )$. Upon substituting the ansatz $(U,V,W)=(u_\textrm{h},v_\textrm{h},w_\textrm{h})(\xi )+(\bar{u},\bar{v},\bar{w})(\xi )e^{i\ell y +\lambda \tau }$ and $c=c_\textrm{h}$, this results in the linear eigenvalue problem

3.5 $$\begin{aligned} \lambda u= &  F_{u}u+F_w w-c_\textrm{h} u_\xi \,,\nonumber \\ \lambda v= &  G_{v}v+G_w w+(1+k-u_\textrm{h}){v}_{\xi \xi }-c_\textrm{h} {v}_\xi -uv_{\textrm{h},\xi \xi }-{u}_{\xi }v_{\textrm{h},\xi }\nonumber \\ &  -u_{\textrm{h},\xi }{v}_{\xi }-\ell ^2(1+k-u_\textrm{h}){v}\,,\nonumber \\ \lambda w= &  H_{v}v+H_w w+\frac{1}{\varepsilon ^2}w_{\xi \xi }-c_\textrm{h} w_\xi -\frac{\ell ^2}{\varepsilon ^2}w \,, \end{aligned}$$

where we have dropped the bars for convenience and we denote $F_{u}(\xi ):=\partial _{u}F(u_\textrm{h}(\xi ), w_\textrm{h}(\xi ))$, etc. We write (3.5) in the form

3.6 $$\begin{aligned} \mathbb {L} (\xi ) \begin{pmatrix} u \\ v \\ w \end{pmatrix}&= \lambda \begin{pmatrix} u \\ v \\ w \end{pmatrix} + \ell ^2 \begin{pmatrix} 0 \\ (1+\kappa -u_\textrm{h})v \\ \frac{1}{\varepsilon ^2} w \end{pmatrix}\,, \end{aligned}$$

where

$$\begin{aligned} \mathbb {L}(\xi ):= \begin{pmatrix}-c_\textrm{h}\partial _\xi + F_{u} & 0 & F_w \\ -v_{\textrm{h},\xi \xi }-v_{\textrm{h},\xi }\partial _\xi & (1+\kappa -u_\textrm{h})\partial _{\xi \xi }-c_\textrm{h}\partial _\xi + G_{v} -u_{\textrm{h},\xi } \partial _\xi & G_w \\ 0 & H_{v} & \frac{1}{\varepsilon ^2}\partial _{\xi \xi }-c_\textrm{h}\partial _\xi +H_w \end{pmatrix}\,. \end{aligned}$$

Due to translation invariance, the derivative $(u,v,w)(\xi ) = (u_\textrm{h}, v_\textrm{h}, w_\textrm{h})'(\xi )$ of the wave with respect to $\xi$ satisfies (3.6) when $\lambda =\ell =0$. To examine long-wavelength interfacial instabilities in the direction transverse to the front propagation, we expand about this solution for small $|\ell |\ll 1$, and noting the symmetry $\ell \rightarrow -\ell$ we obtain

$$\begin{aligned} \lambda _\textrm{c} (\ell )&= \lambda _{\textrm{c},2} \ell ^2 + \mathcal {O}(\ell ^4)\,, \qquad \begin{pmatrix} u(\xi ; \ell ^2) \\ v(\xi ; \ell ^2) \\ w(\xi ; \ell ^2) \end{pmatrix} = \begin{pmatrix} u_{\textrm{h},\xi } (\xi ) \\ v_{\textrm{h},\xi } (\xi ) \\ w_{\textrm{h},\xi } (\xi ) \end{pmatrix}+ \begin{pmatrix} \tilde{u}(\xi ) \\ \tilde{v}(\xi ) \\ \tilde{w}(\xi ) \end{pmatrix} \ell ^2 + \mathcal {O}(\ell ^4)\,, \end{aligned}$$

and substitute into (3.6) to obtain

$$\begin{aligned} \mathbb {L} \begin{pmatrix} \tilde{u}(\xi ) \\ \tilde{v}(\xi ) \\ \tilde{w}(\xi ) \end{pmatrix}&= \lambda _{\textrm{c},2} \begin{pmatrix} u_{\textrm{h},\xi } (\xi ) \\ v_{\textrm{h},\xi } (\xi ) \\ w_{\textrm{h},\xi } (\xi ) \end{pmatrix} + \begin{pmatrix} 0 \\ (1+\kappa -u_\textrm{h})v_{\textrm{h},\xi } (\xi ) \\ \frac{1}{\varepsilon ^2}w_{\textrm{h},\xi } (\xi ) \end{pmatrix}. \end{aligned}$$

This results in the Fredholm solvability condition

3.7 $$\begin{aligned} 0= \Bigg \langle \lambda _{\textrm{c},2}\begin{pmatrix} u_{\textrm{h},\xi } (\xi ) \\ v_{\textrm{h},\xi } (\xi ) \\ w_{\textrm{h},\xi } (\xi ) \end{pmatrix}+ \begin{pmatrix} 0 \\ (1+\kappa -u_\textrm{h})v_{\textrm{h},\xi } (\xi ) \\ \frac{1}{\varepsilon ^2}w_{\textrm{h},\xi } (\xi ) \end{pmatrix}, \begin{pmatrix} u^A (\xi ) \\ v^A (\xi ) \\ w^A (\xi ) \end{pmatrix} \Bigg \rangle _{L^2}\,, \end{aligned}$$

where $(u^A,v^A, w^A)(\xi )$ denotes the unique bounded solution to the adjoint equation

3.8 $$\begin{aligned} \mathbb {L}^A(\xi )\begin{pmatrix} u (\xi ) \\ v (\xi ) \\ w (\xi ) \end{pmatrix}=0\,, \end{aligned}$$

where the adjoint operator $\mathbb {L}^A$ is given by

$$\begin{aligned} \mathbb {L}^A (\xi )&= \begin{pmatrix} c_\textrm{h} \partial _\xi + F_{u} & v_{\textrm{h},\xi } \partial _\xi & 0 \\ 0 & (1+\kappa - u_\textrm{h}) \partial _{\xi \xi } + c_\textrm{h} \partial _\xi + G_{v} - u_{\textrm{h},\xi } \partial _\xi & H_{v} \\ F_w & G_w & \frac{1}{\varepsilon ^2} \partial _{\xi \xi }+c_\textrm{h}\partial _\xi + H_w \end{pmatrix} . \end{aligned}$$

Solving (3.7) for $\lambda _{\textrm{c},2}$, we find

3.9 $$\begin{aligned} \lambda _{c,2}&= - \dfrac{\displaystyle \int _\mathbb {R} \left( 1+\kappa - u_\textrm{h}(\xi )\right) v_{\textrm{h},\xi }(\xi ) v^A(\xi ) + \frac{1}{\varepsilon ^2} w_{\textrm{h},\xi }(\xi ) w^A(\xi ) \textrm{d}\xi }{\displaystyle \int _\mathbb {R} u_{\textrm{h},\xi }(\xi ) u^A(\xi ) + v_{\textrm{h},\xi }(\xi ) v^A(\xi ) + w_{\textrm{h},\xi }(\xi ) w^A(\xi ) \textrm{d} \xi }. \end{aligned}$$

We note that the sign of $\lambda _{\textrm{c},2}$ determines the stability of the interface to transverse long wavelength perturbations. To estimate the expression (3.9), we need to obtain leading-order approximations of the adjoint solution $(u^A,v^A, w^A)(\xi )$.

### Leading order asymptotics of $\lambda _{\textrm{c},2}$

We consider the fast formulation

3.10 $$\begin{aligned} \begin{aligned}&c_\textrm{h}u_\xi +F_{u}(u_\textrm{h}(\xi ),w_\textrm{h}(\xi ))u + v_{\textrm{h},\xi } v_\xi =0\,,\\&(1+\kappa - u_\textrm{h}) v_{\xi \xi }+ c_\textrm{h} v _\xi +G_v(u_\textrm{h}(\xi ),v_\textrm{h}(\xi ),w_\textrm{h}(\xi )) v\\&\quad +H_v(v_\textrm{h}(\xi ),w_\textrm{h}(\xi ))w - u_{\textrm{h},\xi } v_\xi = 0\,, \\&w_{\xi \xi }+ \varepsilon ^2c_\textrm{h} w_\xi +\varepsilon ^2\big ( F_w(u_\textrm{h}(\xi ),w_\textrm{h}(\xi ))u + G_w(u_\textrm{h}(\xi ),v_\textrm{h}(\xi ),w_\textrm{h}(\xi ))v\\&\quad + H_w(v_\textrm{h}(\xi ),w_\textrm{h}(\xi ))w \big ) = 0\,,\\ \end{aligned} \end{aligned}$$

of the adjoint equation (3.8), and the associated slow formulation

3.11 $$\begin{aligned} \begin{aligned}&\varepsilon c_\textrm{h}u_\zeta +F_{u}(u_\textrm{h}(\zeta /\varepsilon ),w_\textrm{h}(\zeta /\varepsilon ))u + \varepsilon ^2 v_{\textrm{h},\zeta } v_\zeta =0\,,\\&\varepsilon ^2 (1+\kappa - u_\textrm{h}) v_{\zeta \zeta }+ \varepsilon c_\textrm{h} v_\zeta +G_v(u_\textrm{h}(\zeta /\varepsilon ),v_\textrm{h}(\zeta /\varepsilon ),w_\textrm{h}(\zeta /\varepsilon )) v\\&\quad +H_v(v_\textrm{h}(\zeta /\varepsilon ),w_\textrm{h}(\zeta /\varepsilon ))w - \varepsilon ^2 u_{\textrm{h},\zeta } v_\zeta = 0 \,,\\&w_{\zeta \zeta }+ \varepsilon c_\textrm{h} w_\zeta +F_w(u_\textrm{h}(\zeta /\varepsilon ),w_\textrm{h}(\zeta /\varepsilon ))u + G_w(u_\textrm{h}(\zeta /\varepsilon ),v_\textrm{h}(\zeta /\varepsilon ),w_\textrm{h}(\zeta /\varepsilon ))v\\&\quad + H_w(v_\textrm{h}(\zeta /\varepsilon ),w_\textrm{h}(\zeta /\varepsilon ))w = 0\,, \end{aligned} \end{aligned}$$

where $\zeta =\varepsilon \xi$, and we abuse notation by writing $u=u(\xi )$ in (3.10), and $u=u(\zeta )$ in (3.11), etc. In the fast field near the interface, to leading order we have $c_\textrm{h}=c_*(w_*)$, $w_\textrm{h}=w_*$, $v_\textrm{h}=v_*(\xi )$, and $u_\textrm{h}=u_*$, where

$$\begin{aligned} c_*(w_*)&={\left\{ \begin{array}{ll}c_1(w_*),& w_*<\delta _1^{-1}\,,\\ c_0(w_*), & w_*>\delta _1^{-1}\,,\end{array}\right. }\\ (v_*,q_*)(\xi ;w_*)&={\left\{ \begin{array}{ll}(v_1,q_1)(\xi ;w_*), & w_*<\delta _1^{-1}\,,\\ (v_0,q_0)(\xi ;w_*), & w_*>\delta _1^{-1}\,,\end{array}\right. }\\ u_*(w_*)&={\left\{ \begin{array}{ll}1-\delta _1w_*, & w_*<\delta _1^{-1}\,,\\ 0, & w_*>\delta _1^{-1}\,.\end{array}\right. } \end{aligned}$$

Thus, to leading order (3.10) becomes

$$\begin{aligned} \begin{aligned} c_*u_\xi +F_{u}(u_*,w_*)u + v_{*,\xi } v_\xi&=0\,,\\ (1+\kappa - u_*) v_{\xi \xi }+ c_* v _\xi +G_v(u_*,v_*(\xi ),w_*) v+H_v(v_*(\xi ),w_*)w&= 0\,, \\ w_{\xi \xi }&= 0. \end{aligned} \end{aligned}$$

Hence *w* is constant to leading order with $w=\bar{w}_*$, and *v* satisfies

3.12 $$\begin{aligned} (1+\kappa - u_*) v_{\xi \xi }+ c_* v _\xi +G_v(u_*,v_*(\xi ),w_*) v&= -H_v(v_*(\xi ),w_*)\bar{w}_*\,. \end{aligned}$$

We define the fast reduced operator

$$\begin{aligned} \mathcal {L}_v:&= (1+\kappa - u_*) \partial _{\xi \xi }- c_* \partial _\xi +G_v(u_*,v_*(\xi ),w_*). \end{aligned}$$

The unique bounded solution of the fast reduced adjoint equation

$$\begin{aligned} \mathcal {L}_v^Av= (1+\kappa - u_*) v_{\xi \xi }+ c_* v _\xi +G_v(u_*,v_*(\xi ),w_*) v&= 0 \end{aligned}$$

is given by $\bar{v}_*(\xi ):=v_{*,\xi }(\xi )e^{-c_* \xi /(1+\kappa -u_*)}$.

Since $v_{*,\xi }$ lies in the kernel of $\mathcal {L}_v$, (3.12) implies that

$$\begin{aligned} 0= \bar{w}_*\int _\mathbb {R} H_v(v_*(\xi ),w_*)v_{*,\xi }(\xi )\textrm{d}\xi = \bar{w}_*\left( H(v_*^+,w_*)-H(0,w_*)\right) = \delta _3\bar{w}_*v_*^+, \end{aligned}$$

where $v_*^+:= v^+(w_*)= \lim _{\xi \rightarrow \infty }v_*(\xi )$ and we recall that $\lim _{\xi \rightarrow -\infty } v_*(\xi )=0$. Hence to leading order $\bar{w}_*=0$ and $v(\xi )=\alpha _*\bar{v}_*(\xi )$. From this we see that *u* satisfies

$$\begin{aligned} c_*u_\xi +F_{u}(u_*,w_*)u + \alpha _*v_{*,\xi } \bar{v}_{*,\xi }(\xi )&=0 \end{aligned}$$

to leading order. Since $F_{u}(u_*,w_*) = 1-2u_*-\delta _1w_* < 0$ (both for $\delta _1 w_* \lessgtr 1$) and since $u(\xi )$ must be bounded, it follows that

$$\begin{aligned} u(\xi )&=\alpha _*\bar{u}_*(\xi ):=-\frac{\alpha _*}{c_*}\int _{\infty }^\xi e^{-\frac{1}{c_*}(1-2u_*-\delta _1w_*) (\xi -s)} v_{*,s}(s) \bar{v}_{*,s}(s)\textrm{d}s. \end{aligned}$$

In the slow fields away from the interface, to leading order

3.13 $$\begin{aligned} \left( u_\textrm{h}(\zeta /\varepsilon ), v_\textrm{h}(\zeta /\varepsilon ), w_\textrm{h}(\zeta /\varepsilon )\right) = \left( u_*(w^\pm (\zeta )),f^\pm (w^\pm (\zeta )), w^\pm (\zeta )\right) , \end{aligned}$$

where $w^-(\zeta )$ denotes the slow orbit of (2.5) on $\mathcal {M}^0_0\cup \mathcal {M}^0_1$ corresponding to $\mathcal {W}^\textrm{u}(0,0)$ and satisfies $w^-(0)=w_*$, and similarly $w^+(\zeta )$ denotes the slow orbit of (2.6) on $\mathcal {M}^+_0\cup \mathcal {M}^+_1$ corresponding to $\mathcal {W}^\textrm{s}(W^+,0)$ and satisfies $w^+(0)=w_*$. Note that whether $w^-$ is contained entirely within $\mathcal {M}^0_1$, or whether $w^+$ is contained entirely within $\mathcal {M}^+_1$, depends on which case we are in, namely, the benign, malignant gap or no-gap cases. To simplify the following computations, we use the analogous notation $f^\pm (w)$ to define the corresponding *v* coordinate along the slow orbits by

$$\begin{aligned} f^-(w)&:= 0, \qquad f^+(w) :=v^+(w), \end{aligned}$$

and we recall that

$$\begin{aligned} u_*(w)&={\left\{ \begin{array}{ll}1-\delta _1w, & w<\delta _1^{-1}\\ 0, & w>\delta _1^{-1}.\end{array}\right. } \end{aligned}$$

In the slow fields, the orbits $w^\pm$ therefore satisfy the existence problem

3.14 $$\begin{aligned} w^\pm _{\zeta \zeta } +H(f^\pm (w^\pm ),w^\pm )&= 0. \end{aligned}$$

Thus, to leading order (3.11) becomes

$$\begin{aligned} \begin{aligned} F_{u}(u_*(w^\pm ),w^\pm )u&=0\,,\\ G_v(u_*(w^\pm ),f^\pm (w^\pm ),w^\pm ) v+H_v(f^\pm (w^\pm ),w^\pm )w&= 0\,, \\ w_{\zeta \zeta }+F_w(u_*(w^\pm ),w^\pm )u + G_w(u_*(w^\pm ),f^\pm (w^\pm ),w^\pm )v + H_w(f^\pm (w^\pm ),w^\pm )w&= 0\,, \end{aligned} \end{aligned}$$

from which we deduce that $u=0$, and

$$\begin{aligned} v&=-\frac{H_v(f^\pm (w^\pm ),w^\pm )}{G_v(u_*(w^\pm ),f^\pm (w^\pm ),w^\pm ) }w\,, \end{aligned}$$

so that *w* satisfies

3.15 $$\begin{aligned} w_{\zeta \zeta } + \left( H_w(f^\pm (w^\pm ),w^\pm )-\frac{H_v(f^\pm (w^\pm ),w^\pm )}{G_v(u_*(w^\pm ),f^\pm (w^\pm ),w^\pm ) }G_w(u_*(w^\pm ),f^\pm (w^\pm ),w^\pm ) \right) w&= 0. \end{aligned}$$

Noting that

$$\begin{aligned} (f^\pm )'(w^\pm )=-\frac{G_w(u_*(w^\pm ),f^\pm (w^\pm ),w^\pm )}{G_v(u_*(w^\pm ),f^\pm (w^\pm ),w^\pm ) }\,, \end{aligned}$$

the equation (3.15) becomes

$$\begin{aligned} w_{\zeta \zeta } + \left( H_w(f^\pm (w^\pm ),w^\pm )+H_v(f^\pm (w^\pm ),w^\pm )(f^\pm )'(w^\pm ) \right) w&= 0, \end{aligned}$$

from which we deduce upon differentiating (3.14) that $w(\zeta ) = \alpha ^\pm w^\pm _\zeta (\zeta )$ in the slow fields.

We recall that in the fast field $\bar{w}_*=0$ to leading order so that $w=\mathcal {O}(\varepsilon )$, and hence in the slow fields we take $w = \varepsilon \bar{\alpha }^\pm w^\pm _\zeta =\bar{\alpha }^\pm w^\pm _\xi$. Since $w^+_\zeta (0)=w^-_\zeta (0)=p_*$, to ensure continuity, we take $\bar{\alpha }^+=\bar{\alpha }^-=\bar{\alpha }$, so that $\bar{w}_*=\varepsilon \bar{\alpha }p_*$. The jump in $w_\xi$ in the slow fields

$$\begin{aligned} \Delta _s w_\xi&= \lim _{\zeta \downarrow 0} w_\xi - \lim _{\zeta \uparrow 0} w_\xi = \bar{\alpha } \left[ \lim _{\zeta \downarrow 0} w^+_{\xi \xi } - \lim _{\zeta \uparrow 0 } w^-_{\xi \xi } \right] = \varepsilon ^2 \bar{\alpha } \left[ \lim _{\zeta \downarrow 0} w_{\zeta \zeta }^+ - \lim _{\zeta \uparrow 0} w_{\zeta \zeta }^- \right] \\&= - \varepsilon ^2 \bar{\alpha } \left[ H(v_*^+, w_*) - H( 0, w_*) \right] = - \varepsilon ^2 \bar{\alpha } \delta _3 v_*^+ \end{aligned}$$

must be accounted for by the change over the fast field

$$\begin{aligned} \Delta _f w_\xi&= -\varepsilon ^2 \alpha _* \int _\mathbb {R} F_w(u_*,w_*)\bar{u}_*(\xi ) + G_w (u_*,v_*(\xi ), w_*) \bar{v}_*(\xi ) \textrm{d} \xi \,, \end{aligned}$$

from which we obtain

3.16 $$\begin{aligned} \frac{\bar{\alpha }}{\alpha _*}&= \frac{\int _\mathbb {R} F_w(u_*,w_*)\bar{u}_*(\xi ) + G_w (u_*,v_*(\xi ), w_*) \bar{v}_*(\xi ) \textrm{d} \xi }{\delta _3 v_*^+}. \end{aligned}$$

We can now estimate (3.9). At leading order

$$\begin{aligned} \int _\mathbb {R} w_{\textrm{h},\xi }(\xi ) w^A(\xi ) \textrm{d} \xi&= \left( \int _{-\infty }^{-\frac{1}{\sqrt{\varepsilon } }} + \int _{-\frac{1}{\sqrt{\varepsilon }} }^{\frac{1}{\sqrt{\varepsilon }}} + \int _{\frac{1}{\sqrt{\varepsilon } } }^\infty \right) w_{\textrm{h},\xi }^*(\xi ) w^A(\xi ) \textrm{d} \xi \\&= \bar{\alpha } \int _{-\infty }^{-\frac{1}{\sqrt{\varepsilon } }} (w_\xi ^-)^2 \textrm{d} \xi + \varepsilon ^2 \bar{\alpha } \int _{-\frac{1}{\sqrt{\varepsilon } }}^{\frac{1}{\sqrt{\varepsilon } }} (q_*)^2 \textrm{d} \xi +\bar{\alpha } \int _{\frac{1}{\sqrt{\varepsilon } }}^\infty (w_\xi ^+)^2 \textrm{d} \xi \\&= \varepsilon \bar{\alpha } \left( \int _{-\infty }^0 (w_\zeta ^-)^2 \textrm{d} \zeta + \int _0^\infty (w_\zeta ^+)^2 \textrm{d} \zeta \right) + \mathcal {O}(\varepsilon \sqrt{\varepsilon } )\,, \end{aligned}$$

and

$$\begin{aligned} \int _\mathbb {R} v_{\textrm{h},\xi }(\xi ) v^A(\xi ) \textrm{d} \xi&= \alpha _* \int _\mathbb {R} v_{*,\xi }(\xi )^2 e^{-c_* \xi /(1+\kappa - u_*)} \textrm{d} \xi + \mathcal {O}(\varepsilon ). \end{aligned}$$

We note that $u_{\textrm{h},\xi }=\mathcal {O}(\varepsilon )$ in the fast field while $u^A(\xi )=0$ to leading order in the slow fields, so by (3.9) and (3.16), we have that

3.17 $$\begin{aligned} \lambda _{c,2}&\sim - \frac{\bar{\alpha }}{\varepsilon \alpha _*} \frac{ \displaystyle \left( \int _{-\infty }^0 (w_\zeta ^-)^2 \textrm{d} \zeta + \int _0^\infty (w_\zeta ^+)^2 \textrm{d} \zeta \right) }{\displaystyle \int _\mathbb {R} v_{*,\xi }(\xi )^2 e^{-\left( \frac{c_*}{1+\kappa - u_*} \right) \xi }\textrm{d} \xi }\nonumber \\&=- \frac{1}{\varepsilon \delta _3 v_*^+}\int _\mathbb {R} F_w(u_*,w_*)\bar{u}_*(\xi ) + G_w (u_*,v_*(\xi ), w_*) \bar{v}_*(\xi ) \textrm{d} \xi \nonumber \\&\quad \times \frac{\displaystyle \left( \int _{-\infty }^0 (w_\zeta ^-)^2 \textrm{d} \zeta + \int _0^\infty (w_\zeta ^+)^2 \textrm{d} \zeta \right) }{\displaystyle \int _\mathbb {R} v_{*,\xi }(\xi )^2 e^{-\left( \frac{c_*}{1+\kappa - u_*} \right) \xi }\textrm{d} \xi }\nonumber \\&= \frac{1}{\varepsilon \delta _3 v_*^+}\frac{\displaystyle \left( \int _{-\infty }^0 (w_\zeta ^-)^2 \textrm{d} \zeta + \int _0^\infty (w_\zeta ^+)^2 \textrm{d} \zeta \right) }{\displaystyle \int _\mathbb {R} v_{*,\xi }(\xi )^2 e^{-\left( \frac{c_*}{1+\kappa - u_*} \right) \xi }\textrm{d} \xi } \left( \int _\mathbb {R} \delta _1u_*\bar{u}_*(\xi ) +\delta _2v_*(\xi ) \bar{v}_*(\xi ) \textrm{d} \xi \right) \end{aligned}$$

at leading order in $\varepsilon$, where

$$\begin{aligned} \bar{v}_*(\xi )&=v_{*,\xi }(\xi )e^{-\frac{c_*}{1+\kappa -u_*}\xi }\\ \bar{u}_*(\xi )&=\frac{1}{c_*}\int ^{\infty }_\xi e^{-\frac{1}{c_*}(1-2u_*-\delta _1w_*) (\xi -s)} v_{*,s}(s) \bar{v}_{*,s}(s)\textrm{d}s. \end{aligned}$$

In particular, the sign of $\lambda _{\textrm{c},2}$ is determined at leading order by

3.18 $$\begin{aligned} \textrm{sign}\left( \lambda _{c,2}\right)&= \textrm{sign}\left( \int _\mathbb {R} \delta _1u_*\bar{u}_*(\xi ) +\delta _2v_*(\xi ) \bar{v}_*(\xi ) \textrm{d} \xi \right) . \end{aligned}$$

In the gap case, $u_*=0$, so that $\lambda _{\textrm{c},2}$ is always positive, provided $\delta _2>0$. However, in the no-gap case, $u_*=1-\delta _1w_*$, so that

$$\begin{aligned} \int _\mathbb {R} \delta _1u_*\bar{u}_*(\xi )&+\delta _2v_*(\xi ) \bar{v}_*(\xi ) \textrm{d} \xi \\&= \int _\mathbb {R}\left[ \frac{\delta _1(1-\delta _1w_*)}{c_*}\int ^{\infty }_\xi e^{-\frac{1}{c_*}(-1+\delta _1w_*) (\xi -s)}v_{*,s}(s) \bar{v}_{*,s}(s)\textrm{d}s\right. \\&\qquad \left. +\delta _2v_*(\xi ) v_{*,\xi }(\xi )e^{-\frac{c_*}{\kappa +\delta _1 w_*}\xi }\right] \textrm{d} \xi . \end{aligned}$$

This term could be positive or negative depending on the relation between $\delta _1, \delta _2$, and the other system parameters $\rho , a, k, \delta _3$.

## Numerical simulations and discussion

The formal geometric singular perturbation analysis of the preceding sections provides a framework by which we can understand the structure of 1D bistable traveling tumor fronts in (1.1), and in particular uncovers geometric mechanisms which distinguish between qualitatively different cases: benign and malignant no-gap/gap tumors. Furthermore, when extending a 1D profile as a straight planar interface in two spatial dimensions, the expression (3.17) provides a stability criterion for long wavelength perturbations in the direction along the interface, assuming that the corresponding traveling wave is stable in one spatial dimension.

While we can deduce from this expression and the arguments in Sect. 3.2 that, for instance, tumor interfaces in the malignant gap case are always unstable in two spatial dimensions when $\varepsilon$ is sufficiently small, in other regimes the sign of the integral expression (3.18) is less apparent due to its implicit dependence on various system parameters. However, we are able to explore different parameter regimes by numerically solving the traveling wave equation (2.1). Figure 8 depicts numerically computed traveling waves profiles for four different sets of parameters. The 1D spectra of the solutions are also shown, implying that these waves are stable in the longitudinal direction, so that when restricted to a one-dimensional domain, small perturbations of the wave decay in time. Also plotted is a continuation of the eigenvalue $\lambda _{\textrm{c,2}}(\ell )$ for small values of the wave number $\ell$ in each case, indicating they are all unstable in two spatial dimensions; we note that each profile exhibits an acellular gap, and hence this matches our theoretical prediction that such solutions are unstable in two dimensions.

**Fig. 9:**
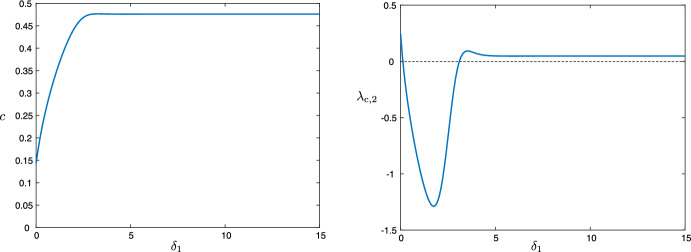
Results of numerical continuation in AUTO07p (Doedel et al. 2007) for the parameter values $(a,\kappa , \delta _2,\delta _3, \rho , \varepsilon ) = (0.1,0.1,0.1,70,1.0,0.0063)$ for values of $\delta _1\in (0.05,15)$: wave speed *c* versus $\delta _1$ (left), $\lambda _{\textrm{c},2}$ versus $\delta _1$ (right)

**Fig. 10:**
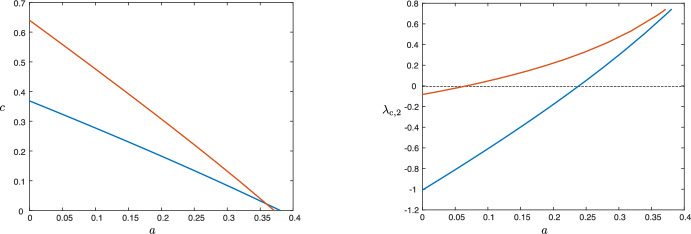
Results of numerical continuation for the parameter values $(\kappa , \delta _2,\delta _3, \rho , \varepsilon ) = (0.1,0.1,70,1.0,$0.0063) for a range of *a*-values for $\delta _1=0.6$ (blue) and $\delta _1=12.5$ (red): wave speed *c* versus *a* (left), $\lambda _{\textrm{c},2}$ versus *a* (right)

Using the expression (3.9), we are also able to track this instability as a function of system parameters. Figure 9 depicts the results of numerical continuation in the parameter $\delta _1$ (the other parameters are the same as the wave in the first row of Fig. 8). Note that while the waves can be 2D stable for smaller values of $\delta _1$, for larger $\delta _1$ the instability appears, matching the prediction of the asymptotic expression (3.17) that the interfaces are unstable in the malignant gap case—the gap appears as $\delta _1$ increases; see Fig. 7. We also note that the speed of the front also increases in $\delta _1$. In Fig. 10, we track the impact of the Allee effect on the coefficient $\lambda _{\textrm{c},2}$. We see that in general, an increase in the Allee effect decreases the speed of the tumor, but also leads to the onset of the long wavelength instability.

**Fig. 11:**
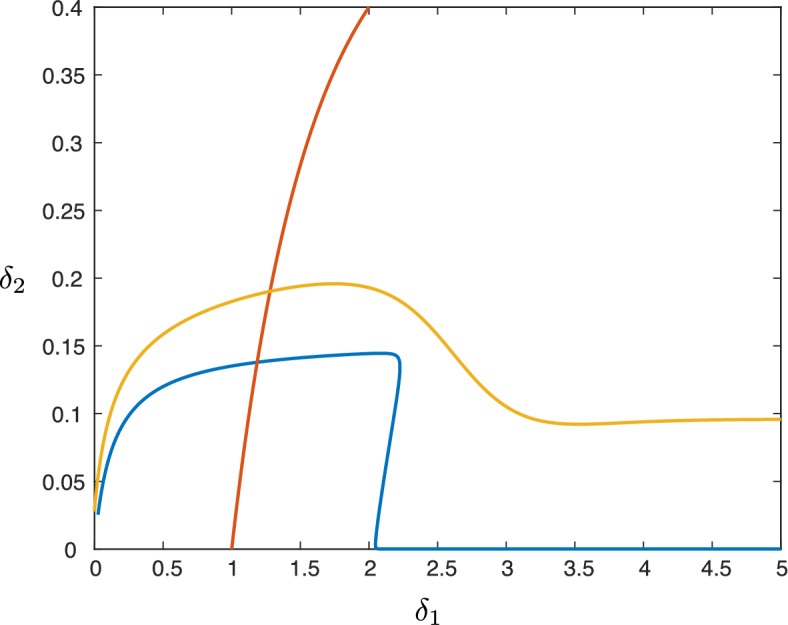
Results of numerical continuation of the curve $\lambda _{\textrm{c},2}=0$ for the parameter values $(a,\kappa , \delta _3, \rho ) = (0.1,0.1,70,1.0)$ in the $(\delta _1,\delta _2)$-plane for $\varepsilon =0.0063$ (yellow) and $\varepsilon =10^{-5}$ (blue). Below each curve we have $\lambda _{\textrm{c},2}<0$, while $\lambda _{\textrm{c},2}>0$ above. Plotted in red is the malignant/benign boundary $\delta _1V_+=1$

By solving numerically for zeros of the expression (3.9), we can also track the stability boundary in parameter space. Figure 11 depicts the stability boundary in $(\delta _1,\delta _2)$-space for two different values of $\varepsilon$. Also shown is the curve which forms the boundary in parameter space between the benign and malignant tumors, which shows that both benign/malignant tumors can be stable/unstable and that in general, the interfaces are more likely to be unstable for larger values of $\delta _1, \delta _2$.

**Fig. 12:**
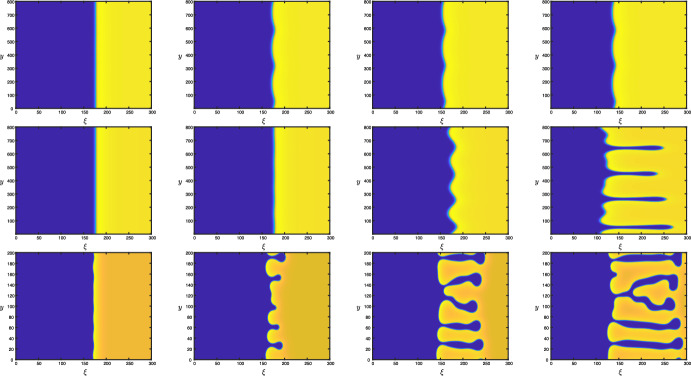
Results of direct numerical simulations exhibiting different manifestations of the long wavelength instability. The simulations were performed in a co-moving frame corresponding to the wave speed of the initial front profile (so that in the absence of any instability, the fronts would appear stationary), with Neumann boundary conditions in $\xi$, and periodic boundary conditions in *y*. Finite differences were used for spatial discretization, and MATLAB’s ode15s routine was used for time stepping. The corresponding *v*-profile is depicted, where yellow indicates high density of tumor cells, and blue depicts low density of tumor cells. (First row) Simulation for the parameter values $(a,\kappa , \delta _1, \delta _2, \delta _3, \rho , \varepsilon ) = (0.25,0.1,12.5,0.1,70.0,1.0, 0.0063)$ at the times $t=5000,10000,15000,20000$ from left to right, with wave speed $c=0.2211$. (Second row) Simulation for the parameter values $(a,\kappa , \delta _1, \delta _2, \delta _3, \rho , \varepsilon ) = (0.35,0.1,12.5,0.1,70.0,1.0, 0.0063)$ at the times $t=10000,20000,30000,40000$ from left to right, with wave speed $c=0.0401$. (Third row) Simulation for the parameter values $(a,\kappa , \delta _1, \delta _2, \delta _3, \rho , \varepsilon ) = (0.25,0.05,11.5,3,1,15, 0.05)$ at the times $t=250,350,450,550$ from left to right, with wave speed $c=0.3296$. In each of the three simulations, initial conditions were constructed by trivially extending the 1D-stable profiles from Fig. 8 (rows 2 through 4) in the *y*-direction and adding a small amount of positive noise

We emphasize that our analysis concerns spectral stability of the traveling fronts, and while these spectral computations may indicate instability, it is not easy to predict the resulting nonlinear behavior of the interface. Nevertheless, direct numerical simulations suggest that planar interfaces are in fact nonlinearly unstable, and the dynamics may lead to complex patterns at the tumor interface. Figure 12 depicts the results of direct numerical simulations for three different choices of the parameters $(a,\varepsilon )$, initialized with 1D-stable traveling wave profiles taken from rows two through four of Fig. 8. Different manifestations of the long wavelength instability appear in each case: in the first simulation the interface develops cusps which remain bounded as the propagation speed of the advancing interface appears to increase, while in the second, the interface breaks up into growing finger patterns; the parameter values for these two simulations were taken from Gatenby and Gawlinski (1996) (with the exception of $(a,\delta _2,\kappa )$, which were not present in Gatenby and Gawlinski (1996)). The third and final simulation exhibits more elaborate growing finger patterns, which develop on a faster timescale. Note that the time taken for the instability to develop varies based on the magnitude of the coefficient $\lambda _{\textrm{c},2}$; see Fig. 8.

From the mathematical point of view, there are a number of further research directions that are directly in line with the present work. More general models also include tumor cell density *V* in the nonlinear diffusion term of the evolution equation for *V* (1.1) and may also contain (nonlinear) diffusion on the *U*-equation. The methods developed here are believed to be sufficiently flexible to investigate more general equations. Within the current framework, several open questions remain. In particular, the basic assumption underlying the two-dimensional stability analysis here (that is numerically validated in several cases, see Fig. 8) is the stability of the front with respect to one-dimensional (longitudinal) perturbations—a rigorous verification of this assumption is the subject of future work. In addition, our (in)stability results are purely at the spectral level, and a challenging direction for future work concerns the study of the nonlinear manifestation of the long wavelength instability studied here.

Our mathematical analysis and numerical simulations naturally lead to a number of biological insights, some of which we now highlight. We showed that the speed of invasion, and the probability of an acellular gap being formed, both increase as $\delta _1$ increases. As this parameter is a measure of the negative impact of lactic acid on normal cells, this result makes intuitive sense. Furthermore, the stronger the Allee effect on the tumor cells, the slower the invasion speed of these cells, a result that also aligns with our intuition. Our study of the front instabilities showed that in such cases (strong Allee effect), the front undergoes a bifurcation that initiates the formation of growing ‘fingers’, resulting in an irregular morphology, while in the case of a weak Allee effect, we predict that the waves will move faster and the initial bifurcation has a milder effect and drives the development of (moving) ‘cusps’ in the front morpholgy, similar to that observed in the experimental results of Gatenby and Gawslinski (1996).

Note that in our model, the instability of the invasion tumor cell front is an emergent property of the system which does not require phenotypic heterogeneity within the tumor cell population or spatial heterogeneity within the system (these are often assumed to be the cases for the break-up of an invading front). However, in reality, tumors typically are very heterogeneous and the extracellular matrix (ECM) through which they move is not spatially homogeneous. Possible future avenues of research would be to extend our present work to include these complications. Again, there is a strong similarity between cancer research and ecology (cf. Korolev et al. (2014)), in which understanding the impact of spatial heterogeneities also is a central issue (cf. Bastiaansen and Doelman (2019) and the references therein). In fact, through the link with ecology, another promising (and challenging) line of research emerges: like in ecology, interfaces between different states—bare soil/vegetated or normal/tumorous—are typically curved, not flat (as assumed here). Thus, it is necessary to extend the current approach to curved interfaces—see Byrnes et al. (2023) for some first steps in that direction in the ecological setting. Note that there also is an important distinction between ecosystem and tumor interfaces: the former typically are within two-dimensional domains (the surface of a terrain), while the latter are intrinsically three-dimensional: in combination with the local curvatures, this additional freedom may for instance have a significant impact on the nature of the protrusions initiated by the fingering instability.

From the modeling point of view, there are also various promising future research directions. For example, Strobl et al. (2020) presents a model of cancer cell invasion in which there are two different tumor cell phenotypes, one producing lactic acid and the other producing proteins that degrade the ECM, considered as a barrier to invasion. They show how different phenotypic spatial structures arise in the invading front depending on the inter-cellular competition dynamics between the two cancer cell phenotypes. More recently, Crossley et al. (2024) present a model based on volume-filling, in which one cancer cell phenotype proliferates, while the other degrades the matrix (an example of the well-known “go-or-grow” hypothesis). Analysis of this model also gives rise to different phenotypically structured invading fronts, depending on parameter values. Both of these studies were carried out in one spatial dimension and it would be interesting to see what structures, in both physical and phenotypic space, they exhibit when considered in two (or three) spatial dimensions. A further modelling complication to address is that, while these models consider the ECM as a barrier to cancer cell invasion, the ECM also enables cell invasion through providing a “scaffold” to which cells can attach and move. Understanding in detail how this dual property of the ECM affects tumor invasion is an open question.

## Data Availability

Simulation data and codes generated during the current study are available from the corresponding author on reasonable request.

## Stability of steady states

We consider

$$\begin{aligned} \begin{aligned} U_\tau&=F(U,W) \,,\\ V_\tau&= G(U,V,W)+\nabla \cdot ((1+\kappa -U)\nabla V)\,,\\ W_\tau&= H(V,W)+\frac{1}{\varepsilon ^2}\Delta W \,, \end{aligned} \end{aligned}$$

where *F*, *G*, *H* are as in (3.2). Linearizing about a steady state $(U,V,W) = (U_0,V_0,W_0)+(\bar{u}, \bar{v}, \bar{w})e^{i(\ell _1 x+\ell _2 y)+\lambda t}$, we obtain the linearized system

A.1 $$\begin{aligned} \lambda \begin{pmatrix} \bar{u} \\ \bar{v} \\ \bar{w}\end{pmatrix}= \begin{pmatrix}F_{u} & 0 & F_w \\ 0 & -(1+\kappa -U_0)(\ell _1^2+\ell _2^2)+ G_{v} & G_w \\ 0 & H_{v} & -\frac{1}{\varepsilon ^2}(\ell _1^2+\ell _2^2) +H_w \end{pmatrix}\begin{pmatrix} \bar{u} \\ \bar{v} \\ \bar{w}\end{pmatrix}\,, \end{aligned}$$

where $F_u: = \tfrac{\partial F}{\partial u}(U_0,V_0, W_0)$, etc. The steady state is stable if all eigenvalues satisfy $\textrm{Re}(\lambda )<0$ for all $\ell _1, \ell _2\in \mathbb {R}$. From this we see that a necessary condition is $F_u<0$, from which we immediately obtain that the steady state $P_1$ is always unstable, while $P_3^+$ is unstable if $\delta _1 V^+>1$ and $P_4^+$ is unstable if $\delta _1 V^+<1$.

The stability of the remaining steady states is determined by the lower right $2 \times 2$ block eigenvalue problem

A.2 $$\begin{aligned} \lambda \begin{pmatrix} \bar{v} \\ \bar{w}\end{pmatrix}= \begin{pmatrix}-(1+\kappa -U_0)(\ell _1^2+\ell _2^2)+ G_{v} & G_w \\ H_{v} & -\frac{1}{\varepsilon ^2}(\ell _1^2+\ell _2^2) +H_w \end{pmatrix}\begin{pmatrix} \bar{v} \\ \bar{w}\end{pmatrix}\,, \end{aligned}$$

which, following Carter et al. (2023), §2.1 are stable for $0<\varepsilon \ll 1$, provided the conditions

A.3 $$\begin{aligned} G_v<0, \qquad G_v+H_w<0, \qquad G_vH_w-G_wH_v>0\,, \end{aligned}$$

are satisfied. Employing these conditions, a short computation shows that $P_2$ is stable, $P_3^+$ is stable if $\delta _1 V^+<1$ and $P_4^+$ is stable if $\delta _1 V^+>1$, and the remaining steady states are unstable.

## Footnotes

1 See Remark 2.1 of Davis et al. (2022) for the rationale behind this non-standard scaling.
